# Susceptibility of prediabetes to the health effect of air pollution: a community-based panel study with a nested case-control design

**DOI:** 10.1186/s12940-019-0502-6

**Published:** 2019-07-15

**Authors:** Yiqun Han, Yanwen Wang, Weiju Li, Xi Chen, Tao Xue, Wu Chen, Yunfei Fan, Xinghua Qiu, Tong Zhu

**Affiliations:** 1 BIC-ESAT and SKL-ESPC, College of Environmental Sciences and Engineering and Centre for Environment and Health, Peking University, Beijing, 100871 China; 2 Peking University Hospital, Peking University, Beijing, 100871 China

**Keywords:** PM_2.5_, Prediabetes, Susceptibility, Cardiometabolic biomarkers, Panel study

## Abstract

**Background:**

Recent studies suggest that people with diabetes or who are at risk of developing diabetes, i.e. prediabetic (preDM), are potentially susceptible to air pollution, but the underlying mechanisms remain unclear because the existing epidemiological studies did not include healthy control groups and only focused on limited health outcomes. We hypothesized that acute exposure to ambient fine particles (PM_2.5_) will lead to enhanced pulmonary and cardiometabolic changes in preDM than healthy individuals.

**Methods:**

We recruited 60 preDM and 60 healthy individuals from a community of 22,343 adults in Beijing China, and arranged each subject to complete up to seven repeated clinical visits with measures of 6 cardiopulmonary biomarkers, 6 cytokines, 4 blood pressure and endothelial function outcomes and 4 glucose metabolism biomarkers.. Moving averaged daily ambient PM_2.5_ in preceding 1–14 days was matched to each subject and the PM_2.5_ associated effect on multiple biomarkers was estimated and compared between PreDM and healthy subjects based on linear mixed effect model.

**Results:**

All the subjects exhibited significant acute elevation of exhaled nitric oxide, white blood cells, neutrophils, interleukin-1α, and glycated haemoglobin with increased exposure to PM_2.5_. PreDM subjects had significant stronger adverse changes compared to healthy subjects in 6 cardiometabolic biomarkers, namely, interleukin-2, interleukin-8, systolic and diastolic blood pressure, augmentation pressure, and glucose. The maximum elevation of these 6 biomarkers in PreDM subjects were 8.6% [CI: 4.1–13.3%], 10.0% [CI: 3.9–16.4%], 1.9% [CI: 0.2–3.6%], 1.2% [CI: − 0.1-2.4%], 5.7% [CI: − 0.1-11.8%], 2.4% [CI: 0.7–4.2%], respectively, per an interquartile increase of ambient PM_2.5_ (61.4 μg m^− 3^) throughout the exposure window of the preceding 1–14 days. No significant difference was observed for the changes in pulmonary biomarkers between the two groups.

**Conclusions:**

PreDM individuals are more susceptible to the acute cardiometabolic effect of air pollution than the healthy individuals. A considerable public health burden can be inferred, given the high prevalence of prediabetes and the ubiquity of air pollution in China and worldwide.

**Electronic supplementary material:**

The online version of this article (10.1186/s12940-019-0502-6) contains supplementary material, which is available to authorized users.

## Introduction

Exposure to ambient fine particulate matter with aerodynamic diameter ≤ 2.5 μm (PM_2.5_) has been widely recognised as a major risk factor for disease burden, and is estimated to contribute to the premature mortality of 4.09 million people worldwide annually [1]. PM_2.5_­associated health effects are not homogeneous among populations, as previous studies have indicated that certain characteristics, including life stage, genetic polymorphisms, and preexisting cardiovascular and respiratory diseases may increase the susceptibility of populations to the health impacts of PM_2.5_ [2].

A growing number of studies over the past two decades have suggested the susceptibility to health effects of ambient particulate matter among those with diabetes or who are at risk of diabetes, i.e. pre-diabetes (preDM) [2]. The global age-standardised prevalence of diabetes rose to 8.8% in 2017 among adults aged 20–79, and has reached 9.7% (114.4 million) in China [3]. PreDM is defined by impaired glucose tolerance and impaired fasting glucose, and the estimated prevalence may reach as high as 50.1% based on a national investigation of 1% of the Chinese population [4]. Every year, 5–10% of people with preDM progress to diabetes [5]. A biological link between preDM and increased susceptibility to the cardiovascular and metabolic effects of PM is plausible, because the onset of diabetes is characterised pathologically by chronic inflammation, metabolic disorder, and progressive deterioration of blood vessels [6].

Early studies reporting higher susceptibility of diabetic patients to the health effects of PM largely focused on health outcomes including mortality [7], cardiovascular disease (CVD)-related hospital admissions [8] and emergency department visits [9–12]. Although results from these studies varied, collectively these observations suggested that diabetic populations experience increased health impacts from PM exposure.

To understand the mechanisms underlying this relationship, a number of studies were conducted to investigate the plausibility of biological pathways such as inflammation, vascular endothelial dysfunction, and increased insulin resistance. These pathways have been well documented in numerous publications as key mechanisms in both the health effects of PM [13] and the course of diabetes progression [6].

Evidence from animal studies has demonstrated enhanced vasoconstriction after PM exposure in aortas from preDM rats [14] and inflammatory activation, metabolic dysfunction, and weight gain in mouse models [15]. A number of epidemiological studies have attempted to examine PM-associated changes in inflammatory biomarkers [16, 17], autonomic function [18], endothelial function [19, 20], blood pressure (BP), and insulin resistance [21] in patients with diabetes or metabolic syndrome. However, these studies did not include control groups and thus could not measure whether diabetic patients are more susceptible to the effects of PM. Only a handful of epidemiological studies have compared PM-associated health effects between diabetic and non-diabetic subjects, and these studies have included a limited set of biomarkers, such as C-reactive protein (CRP), white blood cell (WBC) count [22], von Willebrand factor [23], and vascular reactivity [24]. The evidence thus far is inconsistent and insufficient to determine whether those with diabetes or at risk of diabetes have increased susceptibility to the health effects of PM exposure.

To address the aforementioned limitations and provide convincing evidence of the susceptibility of the preDM population to the health effects of air pollution, we conducted a panel study with a nested case-control design to compare the cardiometabolic and respiratory effects of air pollution exposure on healthy and preDM individuals (SCOPE). The reason for choosing preDM instead of diabetes as our case subjects is that preDM individuals are in the early stage of diabetes onset and are experiencing similar pathological states of chronic inflammation and glucose metabolic disorder, but unlike diabetics, they are not taking hypoglycemic medications that could potentially mask the effects of air pollution on health biomarkers. Additionally, the prevalence of preDM is much higher than that of diabetes in China, which warrants more attention [4]. This report focuses on the comparison of ambient PM_2.5_-associated health effects between preDM and healthy subjects by examining health outcomes related to multiple pathways including inflammation, BP, endothelial function, and glucose metabolism. This work was conducted in Beijing, China, and may be of considerable public health importance given the nationwide preDM epidemic and severe air pollution.

## Materials and methods

The design and protocol of the SCOPE project has been described previously [25]. Briefly, all targeted subjects were recruited from a community-based population (*N* = 22,343) living near the Peking University (PKU) campus. Subjects underwent annual physical examinations at PKU Hospital during 2013. We recruited 60 preDM subjects as a case group and 60 healthy subjects with comparable gender and age distribution as a control group. We aimed to examine air pollution-associated cardiometabolic and respiratory changes within each group and differences in these biological responses between the two groups. In this study, preDM was defined only by the baseline fasting plasma glucose level of 6.1–7.0 mmol/L (i.e. not by impaired glucose tolerance) measured at the latest annual physical examination and 6 months prior to the study commencement. From August 2013 to February 2015, all subjects were randomly scheduled for repeated clinical visits, with at least a 2-month gap between consecutive visits. The study protocol was approved by the Institutional Review Board of the PKU Health Science Centre (IRB00001052–13024), and all subjects provided written informed consent before participating in the study.

### Study population

The 120 subjects completed a total of 589 clinical visits, and we excluded 10 subjects who have completed only one visit in the following analyses. In total, healthy and preDM subjects completed an average of 5.5 and 5.0 clinical visits, respectively. Demographic statistics of healthy and preDM subjects are summarised in Table 1. The age range of the subjects was between 50 and 65, and more female than male subjects (F:M = 63:47) participated in the study. Body mass index (BMI) was calculated for each subject at baseline, and the number of normal weight (BMI < 24), overweight (BMI ≥ 24), and obese (BMI ≥ 30) subjects were 68, 35, and 7, respectively. Three healthy and five preDM subjects were found to be current smokers during the study period; we kept them in the database for the analyses in this study and also performed a sensitivity analysis by removing them. No significant differences in age, gender, BMI, or smoking status were observed between the two groups based on Chi-square test. A higher percentage of preDM subjects than healthy subjects used antihypertensive, lipid-lowering, and anticoagulation medicines (preDM: 23 out of 54, 43.0%; healthy: 7 out of 56, 12.5%).

**Table 1:** Summary of Demographic Characterization and Health Outcomes among Health and PreDM Subjects

		Healthy	PreDM	*p* value
Number of subjects		56	54	
Number of visits		5.5	5.0	
Characteristic	Type			
Age	50–58 years	36	28	0.26
	58–65 years	20	26	
Sex	Female	31	32	0.83
	Male	25	22	
BMI	Normal	36	32	0.83
	Overweight	17	18	
	Obese	3	4	
Smoker	No	53	49	0.67
	Yes	3	5	
Medication	No	49	31	< 0.01
	Yes	7	23	
Health Outcomes	Unit			
FE_NO_	ppb	22.0 (15.3)	22.6 (16.0)	0.92
WBC	10^9^/L	5.6 (1.5)	5.9 (1.3)	0.02
Neut	10^9^/L	3.3 (1.1)	3.3 (1.0)	0.17
Mono	10^9^/L	0.3 (0.1)	0.3 (0.1)	0.95
Lymph	10^9^/L	1.9 (0.5)	2.0 (0.6)	< 0.01
CRP	mg/L	0.1 (0.2)	0.2 (0.3)	< 0.01
TNF (Serum)	pg/ml	5.9 (3.7)	5.6 (5.9)	0.08
IL1-α (Serum)	g/ml	2.3 (3.8)	1.9 (2.6)	0.15
IL1-β (Serum)	pg/ml	6.5 (3.0)	6.3 (3.9)	0.60
IL-2 (Serum)	pg/ml	11.3 (1.8)	12.2 (9.0)	0.50
IL-6 (Serum)	pg/ml	5.3 (3.0)	10.0 (34.9)	0.78
IL-8 (Serum)	pg/ml	15.3 (11.4)	17.4 (14.2)	< 0.01
Glu	mmol/L	5.6 (0.5)	6.5 (1.1)	< 0.01
Ins	uU/mL	6.3 (3.8)	8.2 (4.8)	< 0.01
HOMA-IR	mmol*uU	1.6 (1.0)	2.4 (2.0)	< 0.01
HbA1c	%	5.5 (0.3)	6.0 (0.8)	< 0.01
SBP	mmHg	111.9 (10.6)	117.6 (11.2)	< 0.01
DBP	mmHg	78.9 (7.3)	81.6 (7.7)	< 0.01
AP75	%	7.7 (4.0)	8.8 (3.5)	< 0.01
RHI	NA	1.9 (0.6)	1.9 (0.6)	0.78

The statistical analysis for the difference of demographical characterizations between healthy and PreDM subjects were based on Chi-square test; The summary of the 20 biomarkers were described with the format of mean (SD), and the statistical analysis for the difference between healthy and PreDM subjects were based on ANOVA analysis

*Abbreviations*: *BMI* Body mass index, *FE*_*NO*_ Fractional exhaled nitric oxide, *WBC* White blood cell, *Neut* Neutrophil, *Mono* Monocytes, *Lymph* Lymphocyte, *CRP* C-reactive protein, *TNF* tumor necrosis factor–α, *IL* Interleukin, *Glu* Fasting glucose, *Ins* Insulin, *HOMA-IR* Homeostatic model assessment of insulin resistance, *HbA1c* Hemoglobin A1c, *SBP* Systolic blood pressure, *SDP* Diastolic blood pressure, *AP75* Heart rate of 75 pulse beats, *RHI* Reactive hyperemia index

### Health outcomes

All subjects were instructed to fast for at least 8 h before coming for clinical visits, and biological samples were collected between 8 and 10 a.m. on the visiting day. This study focused on four categories of biological changes and measured a total of 20 health outcomes, namely, 1) respiratory and cardiovascular inflammatory biomarkers, including fractional exhaled nitric oxide (FE_NO_), plasma C-reactive protein (CRP), blood cell counts of total white blood cells (WBCs), neutrophils, monocytes, and lymphocytes; 2) cytokines in serum as systemic inflammatory biomarkers, including tumour necrosis factor alpha (TNF-α), interleukin (IL)-1α, IL-1β, IL-2, IL-6, and IL-8; 3) systolic and diastolic BP as well as endothelial function indicators, including aortic augmentation pressure normalised to a heart rate of 75 pulse beats (AP75) and reactive hyperemia index (RHI); 4) glucose metabolism biomarkers, including fasting glucose, insulin, HOMA-IR (homeostatic model assessment of insulin resistance, calculated based on fasting glucose and insulin), and glycated haemoglobin (HbA1c). A summary of the health outcomes and related measurement approaches is listed in Additional file 1: Table S1 and in a previous paper [25].

### Ambient air pollution

Ambient air pollution was measured at a fixed monitoring station on the roof of a six-floor building on the campus of PKU [26], namely, the Peking University Urban Atmosphere Environment Monitoring Station (PKUERS). Among the 120 subjects, 80 (67.5%) participants resided within 5 km of PKUERS, 4 (0.3%) lived more than 10 km away, and the rest were in between. Multiple air pollutants were measured during the campaign, including gaseous pollutants and the size distribution and chemical composition of particulate matter, but in this paper we focused on the association between PM_2.5_ mass concentration and health outcomes. Daily PM_2.5_ samples were collected on Teflon filters using a particulate sampler (TH 16A, Wuhan Tianhong Instruments Co., Ltd., Hubei, China) with a flow rate of 16.7 L min^− 1^. Sampling commenced at 8 a.m. and ended at 7:30 a.m. the following day during the whole panel study period. Blank and sampled filters were weighed with an analytical balance (Mettler Toledo AX105DR, detection limit of 10 μg) in a super clean lab [20 ± 1 °C; relative humidity 40 ± 3%].

### Statistical analysis

Descriptive statistics of exposure to ambient PM_2.5_ and health outcomes were calculated for all subjects by case and control groups, and between-group comparisons were performed using ANOVA analysis with random effect. The moving average of ambient PM_2.5_ concentration during the 1–14 days prior to the clinical visit was linked with the health outcome measurement for each subject as an indicator of cumulative exposure. These moving average concentrations are referred to as “average 1- to 14-day exposure to PM_2.5_” in the following context: e.g. “average 7–day exposure to PM_2.5_” refers to the mean concentration of ambient PM_2.5_ in the preceding 1–7 days before measurement of health outcomes. This time window was chosen because previous studies have suggested that most acute to sub-acute effects of PM_2.5_ on study outcomes can be observed during this time period [13].

To assess the associations between exposure to ambient PM_2.5_ and each biomarker, we applied a linear mixed effects model that included a single random intercept for participants and assumed equal correlation between all observations within participants. Multiple variables were controlled for in the model, including age, sex, BMI, smoking status, medication usage, disease history, and day of the week (DOW). Temperature and RH were also adjusted with a natural splines function with four degrees of freedom, determined by the minimum Akaike information criterion (AIC). To investigate the differences in biological responses to ambient PM_2.5_ between preDM subjects and healthy controls, an interaction term of exposure and group was included in the model. For all analyses, we investigated the short-term effects of air pollution with the exposure windows during the preceding 1–14 days before clinical visits.

All statistical analyses were performed using R statistical software (www.r-project.org). A two–tailed P-value < 0.05 was considered statistically significant in the main context. Considering the multiple health outcomes in this study, we also adjusted P-value based on the Benjaminiand-Hochberg method as a secondary analysis false discovery rate correction [27].

## Results

Descriptive statistics for 20 biomarkers among the 56 healthy and 54 preDM subjects are summarised in Table 1 to portray their biological status. Compared with healthy subjects, preDM subjects exhibited significantly higher levels of WBCs, blood lymphocytes, CRP, fasting glucose, insulin, HOMA-IR, systolic and diastolic BP, augmentation pressure, and serum IL-8.

**Fig. 1:**
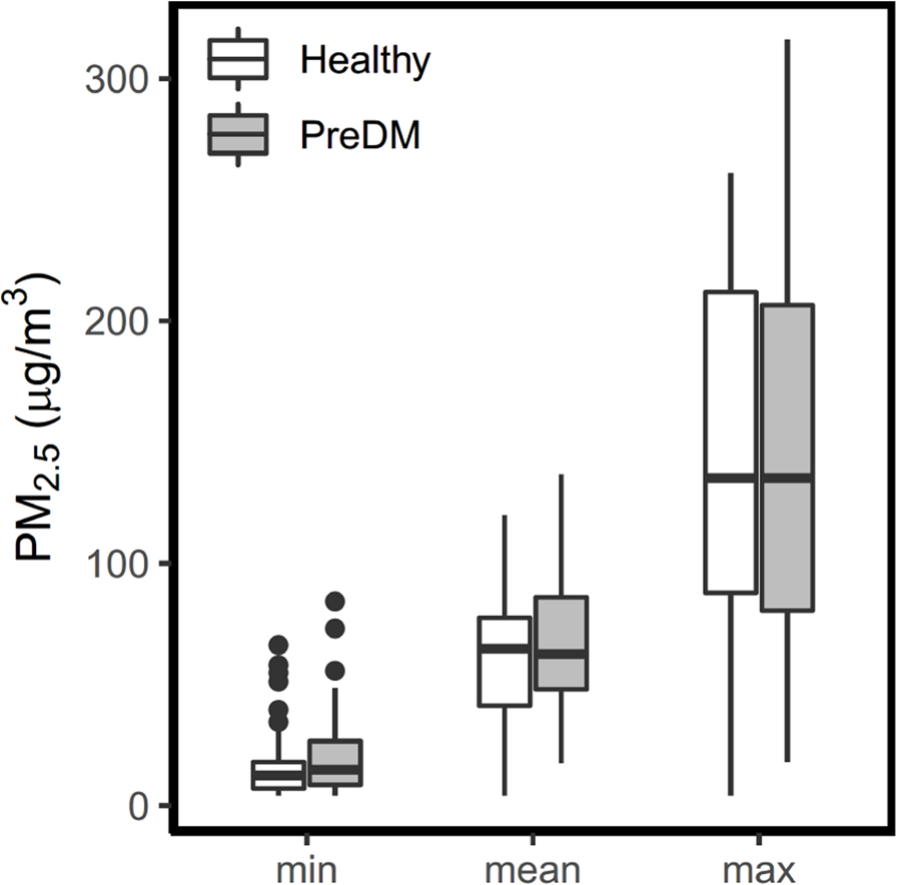
Summary of Average 1-day exposure to PM_2.5_ in healthy and pre-diabetic subjects

Figure 1 shows the minimum, average, and maximum concentrations of average 1-day exposure to PM_2.5_ for 54 preDM and 56 healthy groups. Across the repeated clinical visits, the mean (standard deviation, SD) of average 1-day PM_2.5_ concentration that preDM subjects were exposed to was 69.6 (29.4) μg m^− 3^, and for healthy subjects exposure was 63.2 (27.2) μg m^− 3^. No significant difference in average 1-day exposure was found between the two groups (*p* = 0.47), or in cumulative average 1- to 14-days exposure (Additional file 1: Table S2).

A large range of PM_2.5_ exposure was observed throughout multiple clinical visits for each subject (Fig. 1). On average, the range (difference between maximum and minimum) of exposure to ambient PM_2.5_ experienced by each subject was 129.7 (81.9) μg m^− 3^ in preDM subjects (92.6% of which was over 40.0 μg m^− 3^), and 131.0 (76.0) μg.m^− 3^ in healthy controls (94.5% of which was over 40.0 μg.m^− 3^).

**Fig. 2:**
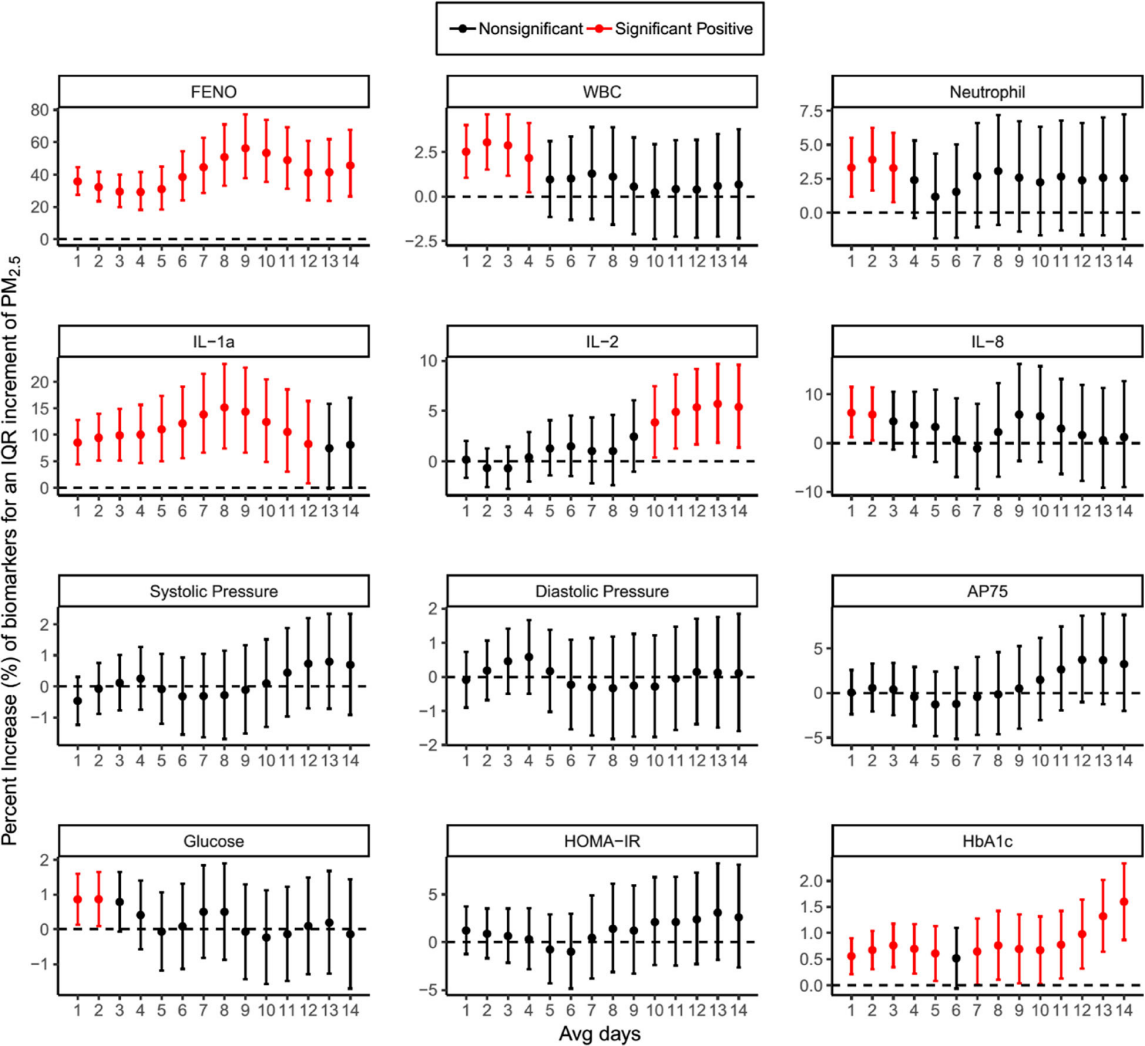
Ambient PM_2.5_-associated effects on the biological changes in cardiorespiratory and metabolic biomarkers in all subjects

Figure 2 shows the biological changes in 12 indicator biomarkers per an interquartile range (IQR) increase in the average 1- to 14-day exposure to ambient PM_2.5_ within all subjects. Each horizontal panel of Fig. 2 displays the changes of three biomarkers from each of the four outcome categories as representatives. The results of the other biomarkers were listed in Additional file 1: Figure S1. The colours red and black indicate significant positive and nonsignificant associations, respectively. Different health outcomes varied in their responses to PM_2.5_ exposure. For instance, an IQR increase in ambient PM_2.5_ (61.4 μg m^− 3^) was associated with a significant elevation in FE_NO_ ranging from 29.3% [confidence interval (CI): 18.1–41.4%] to 56.3% [CI: 37.8–77.2%] throughout the exposure window of the preceding 1–14 days. A significant increase was also observed in inflammatory cells and cytokines, with maximum elevation of 3.0% [CI: 1.5–4.6%], 3.9% [CI: 1.6–6.2%], 15.2% [CI: 7.5–23.4%], 5.7% [CI: 1.9–9.7%], 6.2% [CI: 1.2–11.5%] for WBCs, neutrophils, IL-1α, IL-2, and IL-8 respectively in different exposure time windows. No significant changes were observed for BP, endothelial function, and glucose metabolism, except for elevated fasting glucose and HbA1c with maximum increase of 0.9% [CI: 0.1–1.6%] and 1.6% [CI: 0.9–2.3%] respectively.

**Fig. 3:**
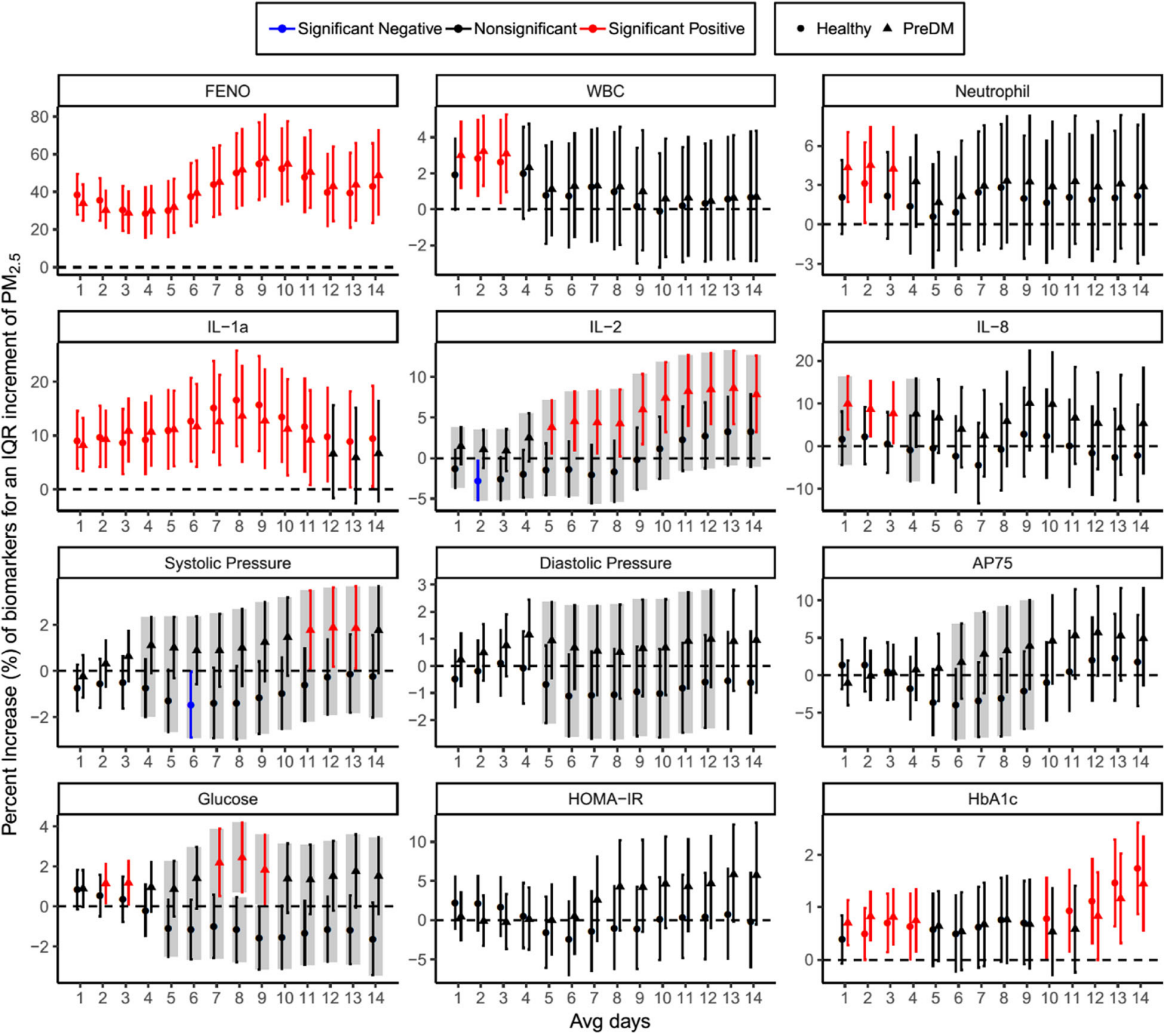
Ambient PM_2.5_-associated cardiorespiratory and metabolic changes in healthy and preDM subjects

Figure 3 showed the comparative results of the ambient PM_2.5_ associated adverse effect between healthy and preDM subjects for the selective 12 biomarkers (the other 8 biomarkers were shown in Additional file 1: Figure S2). The colours red, black, and blue indicate significant positive, nonsignificant, and significant negative associations, respectively. The shaded box identifies the significant difference between preDM and healthy subjects. Out of the total 20 biomarkers, we observed significant enhanced cardiometabolic changes in PreDM than healthy subjects for 6 biomarkers, namely, IL-2, IL-8, systolic BP, diastolic BP, AP75, and glucose in comparable time windows. Specifically, among PreDM subjects, the maximum increases in IL-2, IL-8, systolic BP, diastolic BP, AP75, and glucose throughout the exposure window of the preceding 1–14 days are 8.6% [CI: 4.1–13.3%], 10.0% [CI: 3.9–16.4%], 1.9% [CI: 0.2–3.6%], 1.2% [CI: − 0.1-2.4%], 5.7% [CI: − 0.1-11.8%], 2.4% [CI: 0.7–4.2%], respectively. In contrast, the biological changes in the 6 biomarkers in healthy subjects was statistically nonsignificant throughout exposure window. No significant difference was observed for the changes in the pulmonary biomarker (i.e.FE_NO_) between the two groups.

Sensitivity analysis was also performed by removing the eight smokers from our analyses, and no significant differences were found in the results, as shown in Additional file 1: Figures S3, S4, S5 and S6.After the false discovery rate correction, the PM_2.5_-associated effect on the biomarkers among all the subjects were basically unchanged with similar lag pattern (Additional file 1: Figures S7 and S8).

## Discussion

In this community-based panel study with nested case-control design, we observed significant differences between preDM and healthy subjects in biological changes after short-term exposure to ambient PM_2.5_. Both preDM and healthy subjects had significant elevations in FE_NO_, WBCs, neutrophils, IL-1α, IL-2, IL-8, and HbA1c with increased exposure to PM_2.5_ in comparable time windows. Compared to healthy subjects, preDM subjects showed significantly enhanced PM_2.5_-associated systemic inflammation (increased serum IL-2 and IL-8), elevated systolic and diastolic BP, impaired endothelial function (increased AP75), and elevated fasting glucose levels.

Early evidence of the susceptibility of diabetic patients to air pollution was mostly based on time series studies, and collectively argued for a potential increased short-term impact of PM on CVD-related morbidity [9, 10] and mortality [7, 8]. For example, an analysis of Medicare data from four U.S. cities suggested that diabetic patients have double the risk of PM-mediated cardiovascular hospital admissions compared with nondiabetic patients [9]. A study in Canada with a mean PM_2.5_ concentration of 32 μg m^− 3^ also observed a significant positive association between daily mortality and 3-day mean ambient PM_2.5_ in diabetic patients [7]. To explain this increased susceptibility, a number of epidemiological studies examined potential pathways in diabetic participants, such as inflammation [17], vascular endothelial dysfunction [19, 20], elevated BP, and insulin resistance [21]. These proposed mechanisms are largely based on the assumption that during the onset of diabetes, individuals are characterised by pathological chronic inflammation, blood vessel deterioration, and glucose metabolic disorder [6], which provide a biologically plausible mechanism by which diabetes may aggravate PM-associated cardiovascular and metabolic impacts.

Inflammation is known as a key pathway in both PM-induced adverse health effects [13] and diabetes-related disorders [6]. It involves complicated changes in the levels of inflammatory cells and molecules. The significant increases in FE_NO_, WBCs and neutrophils in both groups after acute exposure to ambient PM_2.5_ observed in this study confirm this classic mechanism of immune activation [28] in both respiratory and cardiovascular systems, which may also be associated with alterations in BP, endothelial function, and insulin resistance [29]. However, the responses of multiple cytokines in our study varied, and do not yield clear conclusions. For example, we observed PM_2.5_-associated elevations in some pro-inflammatory cytokines (i.e. IL-1α, IL-2, and IL-8), but we were surprised to observe significant decreases in TNF-α and IL-6 (Additional file 1: Figure S2), two major proinflammatory cytokines reported in previous studies [13]. Additionally, we did not observe differences in inflammatory responses between preDM and healthy subjects, except in IL-2 and IL-8. Only one study by Dubowsky et al. [22] has investigated how diabetic (*n* = 8) vs. non-diabetic (*n* = 36) subjects differed in inflammatory responses to air pollution, and they reported a significant enhancement in CRP and IL-6 levels in diabetic patients. The differences between Dubowsky’s study [22] and ours may be attributed to differences between diabetic and preDM subjects, the sample sizes in the two studies, and also the complicated inherited molecular mechanisms of inflammation.

In terms of interpreting the BP response to PM, although many studies support that PM or diesel exhaust increase arterial BP, the evidence thus far remains insufficient for a clear conclusion [30]. Inconsistency among previous studies may be partly due to the choice of study subjects, as most of them were healthy subjects. A recent study by Brook et al. [21] focused on potentially susceptible subjects with metabolic syndrome and insulin resistance, and observed a significant 2.0–2.7 mmHg increase in SBP per 67.2 μg m^− 3^ increase in ambient PM_2.5_ (average 1–7 days). Our study further confirmed that pattern, as we observed a − 0.2–0.8% increase in SBP (equal to − 0.3 to 1.0 mmHg) per 56 μg m^− 3^ increase in PM_2.5_ in preDM subjects, and a statistically nonsignificant change in healthy subjects. Considering the potential confounding effect of hypertensive status and medication, we had further run the examinations excluding subjects with high blood pressure (PreDM =18, Healthy = 4). The differences in the PM_2.5_-associated effect on BP elevation between the two preDM and healthy subjects remained significant with similar lag pattern (Additional file 1: Figure S9). Together with Brook’s study, our results support that the progression of glucose metabolic disorder may aggravate the PM-induced elevation in arterial BP. Although the underlying mechanism remains unclear so far, based on the emerging evidence from both epidemiologic and experimental studies, the interaction of glucose metabolic disorder and hypertension may relate to their mutual biological pathways, such as systemic inflammation, oxidative stress, and imbalanced central nervous system [31].

Endothelial dysfunction may be another critical pathway explaining susceptibility to PM-associated health effects in diabetic and preDM subjects [32]. An animal study reported that aortas from pre-diabetic rats were more susceptible to repeated exposures to oil combustion particles in terms of vasomotor change [14]. Some epidemiological studies have also observed a PM_2.5_-associated decrement in flow mediated dilatation, and found greater effects in diabetics than in those at risk [24], and among participants with high haemoglobin levels [19]. In the last decade, noninvasive biomarkers such as augmentation index and RHI were also developed to evaluate vessel stiffness and peripheral endothelial dysfunction, and were supported as promising predictors for CVD events and target organ damage [33]. A recent study applied these measurements to evaluate the ambient PM_2.5-_associated endothelial responses in patients with metabolic syndrome and insulin resistance; however, PM_2.5_ was only mildly associated with augmentation index and not significantly associated with RHI [21]. Our study also showed nonsignificant changes in augmentation pressure and RHI, although adverse changes were slightly higher in preDM than in healthy subjects.

The evidence for potential links between air pollution and the onset of diabetes has increased rapidly in the past decade [34, 35]. Although the underlying biological mechanisms remain unclear, some animal studies suggest that insulin resistance, glucose homeostasis [36], and pulmonary and systemic inflammation [32] are involved. These proposed mechanisms are also supported by cross-sectional studies. For example, the annual PM exposure in Taiwan was reported to be associated with increased fasting glucose and HbA1c levels in younger adults and the elderly [37, 38]. Limited recent studies have also investigated the acute and subacute effects of PM exposure on glucose metabolism, and observed increased HOMA-IR in subjects with metabolic syndrome [21] and elevated blood glucose and HbA1c in healthy subjects [39]. Our study further supports the potential effects of short-term PM_2.5_ exposure on glucose metabolic disorder but suggests that significant elevated glucose and marginally significant elevated HOMA-IR occur in preDM but not healthy subjects. Given the paucity of evidence in this area, more research is needed to confirm and understand the short-term effects of PM exposure on glucose metabolic changes, especially in humans.

To our knowledge, this is the first study to examine the susceptibility of preDM subjects to the health effects of air pollution. By comparing PM_2.5_-associated effects between preDM and healthy subjects, we provide direct evidence that preDM patients, who are in the early stages of diabetes but make up a much larger population than diabetic patients, already have enhanced risks of air pollution-associated health impacts. Additionally, by closely examining a rich set of health outcomes, we found that this enhanced impact manifested in multiple cardiovascular and metabolic pathways, which might support a synergistic effect of air pollution and glucose metabolic disorder on increased propensity for CVD and DM.

Some limitations are worth noting. First, all of the subjects in SCOPE were aged 50–65 years and were of relatively high socioeconomic status; therefore, more comparative studies are needed to extend our findings on the susceptibility of preDM subjects to air pollution to a broader population with a wider range of characteristics. Second, although this study included a great number of cardiovascular and metabolic biomarkers, our knowledge of the short term effects of air pollution on certain pathways remains largely insufficient and warrants more attention, especially for endothelial dysfunction and glucose metabolism disorder. Third, to clearly demonstrate health impacts on multiple pathways, this study focused on exposure to the ambient pollutant of greatest concern, i.e. PM_2.5_ mass concentration. Further analyses will follow in the next stage to elucidate the sizes and critical chemical species of PM_2.5_ or sources among a range of air pollutant mixtures.

## Conclusions

Our study provides evidence that the pre-diabetic subjects is susceptible to the cardiometabolic and inflammatory impacts of air pollution. A considerable public health burden can be inferred, given the high prevalence of prediabetes and the ubiquity of air pollution in China and worldwide. More attention is needed to understand the biological mechanisms and to develop effective interventions.

## Additional file

Additional file 1: Supplemental material. (DOCX 3 kb)

## Data Availability

The datasets generated and/or analysed during the current study are not publicly available due to the requirement of project but are available from the corresponding author on reasonable request.
