# Supplementary material for: Susceptibility of prediabetes to the health effect of air pollution: a community-based panel study with a nested case-control design
Source: Environ Health. 2019 Jul 15;18:65. doi: 10.1186/s12940-019-0502-6 (PMC6631920; doi:10.1186/s12940-019-0502-6)
Supplement: Supplementary file 1 — Supplemental material. (DOCX 3 kb) [file 12940_2019_502_MOESM1_ESM.docx]

**SUPPLEMENTAL MATERIAL**

Yiqun Han^1#^, Yanwen Wang^1#^, Weiju Li^2^, Xi Chen^1^, Tao Xue^1^, Wu Chen^1^, Yunfei Fan^1^, Xinghua Qiu^1^, Tong Zhu^1^*

**Introduction:**

The colours red, black, and blue in Figures S1-9 indicate significant positive, non-significant, and significant negative associations, respectively. To be noted, for Figure S7 and S8, the colours are based on FDR corrected p-value. The shaded box in Figure S5 and S9 identifies the significant difference between preDM and healthy subjects.

**Table S1: Summary of the health outcomes used in this study**

| **Categories** | **Sample** | **Method/Device** | **Health Endpoints** |
| --- | --- | --- | --- |
| **Respiratory and Cardiovascular Inflammation** | Exhaled Breath | Chemiluminescence NOx analyzer (model 42i; Thermo Scientific) | FE_NO_ |
|  | Serum | Automated Nephelometry | CRP |
|  | Plasma | Sysmex XE-2100 Automated Hematology System | WBC/neutrophil/monocyte/lymphocyte |
| **Systemic Inflammatory Cytokines** | Serum | Flow cytometer (Becton Dickinson, USA),  Cytometric Bead Array (CBA) method | IL1α/IL1β/IL2/IL6/IL8/TNF-α |
| **Blood Pressure and Endothelial Function** | NA | Mercurial Sphygmomanometer | Systolic Pressure/Diastolic Pressure |
|  | NA | SphygmoCor (AtCor Medical, Sydney, Australia) | AP75 |
|  | NA | EndoPat 2000 (Itamar Medical, Ltd., Caesarea, Israel) | RHI |
| **Glucose Metabolism** | Serum | Olympus AU2700 biochemical analyzer | Glucose/Insulin/HOMA-IR |
|  | Plasma | Bio-Rad Variant II Turbo analyzer | HbA1c |

**Table S2: Cumulative exposure of PM_2.5_ with Avg 1– to 14– days**

**among healthy and PreDM subjects**

| **Avg** | **Mean** | **Sd** | **Median** | **IQR** |  | **Mean** | **Sd** | **Median** | **IQR** | ***P.value***  **t-test** | ***P.value***  **wilcox** |
| --- | --- | --- | --- | --- | --- | --- | --- | --- | --- | --- | --- |
|  | **Health** | | | |  | **PreDM** | | | |  |  |
| 1 | 63.2 | 27.2 | 64.7 | 36.3 |  | 69.6 | 29.4 | 62.5 | 38.1 | 0.24 | 0.47 |
| 2 | 60.5 | 20.9 | 60.2 | 25.9 |  | 68.9 | 25.9 | 65.8 | 41.0 | 0.07 | 0.17 |
| 3 | 61.2 | 17.1 | 61.4 | 22.6 |  | 69.5 | 24.1 | 66.5 | 33.9 | 0.04 | 0.12 |
| 4 | 61.5 | 15.3 | 60.9 | 22.1 |  | 67.9 | 21.3 | 65.7 | 30.1 | 0.07 | 0.24 |
| 5 | 62.7 | 13.5 | 62.7 | 16.1 |  | 67.4 | 17.6 | 66.8 | 25.3 | 0.12 | 0.27 |
| 6 | 64.5 | 12.0 | 65.0 | 15.2 |  | 67.3 | 16.5 | 65.5 | 24.2 | 0.32 | 0.59 |
| 7 | 66.2 | 10.3 | 66.9 | 14.2 |  | 67.8 | 15.6 | 65.0 | 24.5 | 0.53 | 0.90 |
| 8 | 66.9 | 10.6 | 68.2 | 13.8 |  | 68.0 | 14.8 | 67.1 | 21.4 | 0.66 | 0.95 |
| 9 | 68.5 | 9.6 | 69.1 | 12.8 |  | 67.4 | 13.4 | 67.0 | 17.6 | 0.62 | 0.48 |
| 10 | 69.5 | 10.0 | 70.3 | 14.3 |  | 66.5 | 12.5 | 65.0 | 16.1 | 0.17 | 0.10 |
| 11 | 69.9 | 9.3 | 71.1 | 13.3 |  | 66.8 | 12.5 | 66.5 | 14.9 | 0.15 | 0.08 |
| 12 | 69.9 | 8.4 | 71.1 | 11.1 |  | 67.8 | 13.1 | 67.0 | 15.6 | 0.33 | 0.19 |
| 13 | 69.9 | 8.5 | 70.7 | 11.6 |  | 68.7 | 13.1 | 69.4 | 16.2 | 0.58 | 0.48 |
| 14 | 70.3 | 8.7 | 70.3 | 11.6 |  | 68.4 | 12.8 | 70.3 | 15.3 | 0.37 | 0.41 |

**Figure S1: Ambient PM_2.5_ associated effect on monocytes, lymphocytes, CRP, Interlukin-1β, Interlukin-6, RHI and insulin in all the healthy and preDM subjects.**

**Figure S2: Comparison of Ambient PM_2.5_ associated effect on monocytes, lymphocytes, CRP, Interlukin-1β, Interlukin-6, RHI and insulin between the healthy and preDM subjects.**


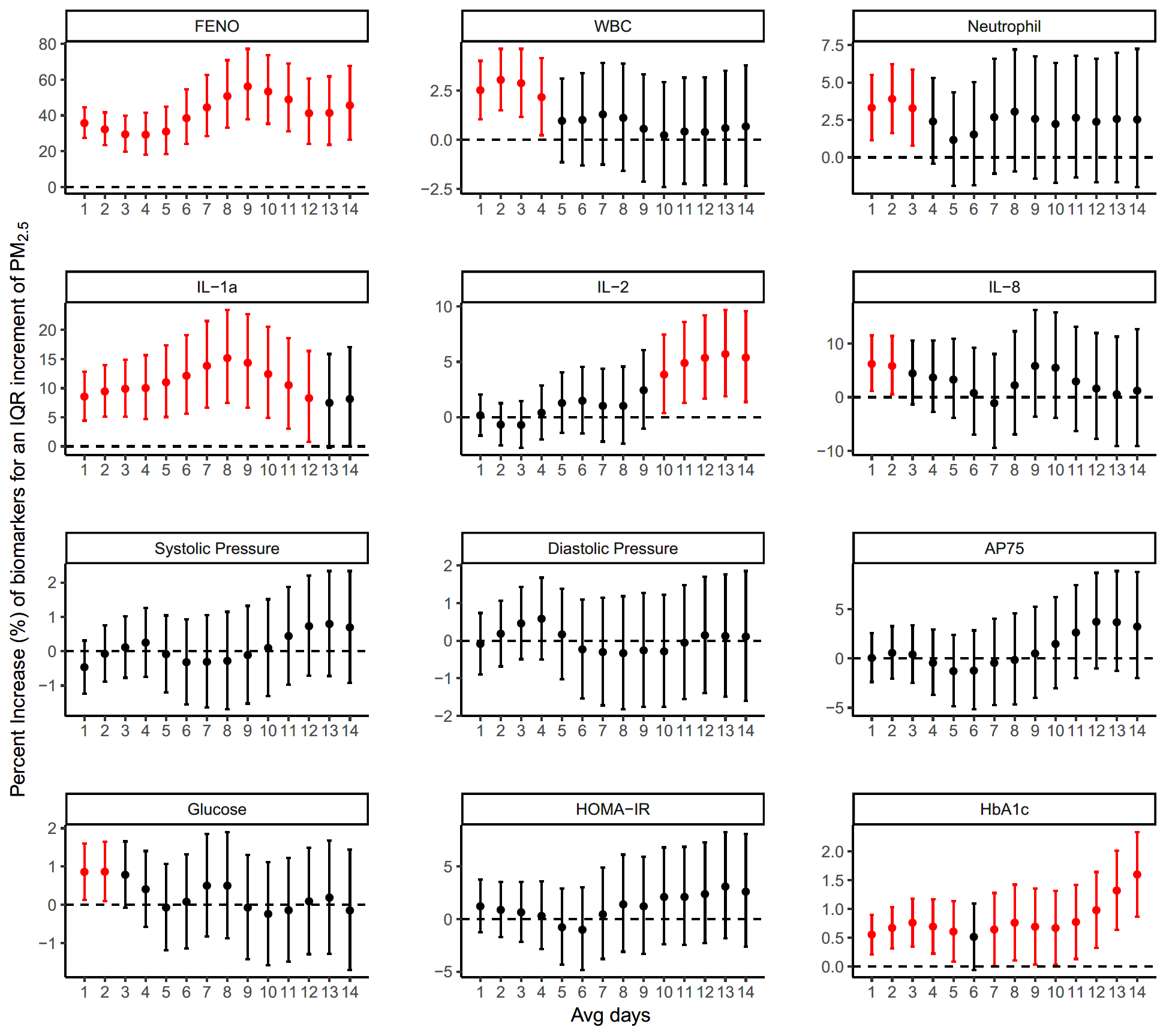


**Figure S3: Sensitivity analysis in nonsmokers: The ambient PM_2.5_ associated effect on 12 biomarkers (eNO, WBC, Neutrophil, Interlukin-1α, Interlukin-2, Interlukin-8, SBP, DBP, AP75, fasting glucose, HOMA-IR, and HbA1c) in all the healthy and preDM subjects.**


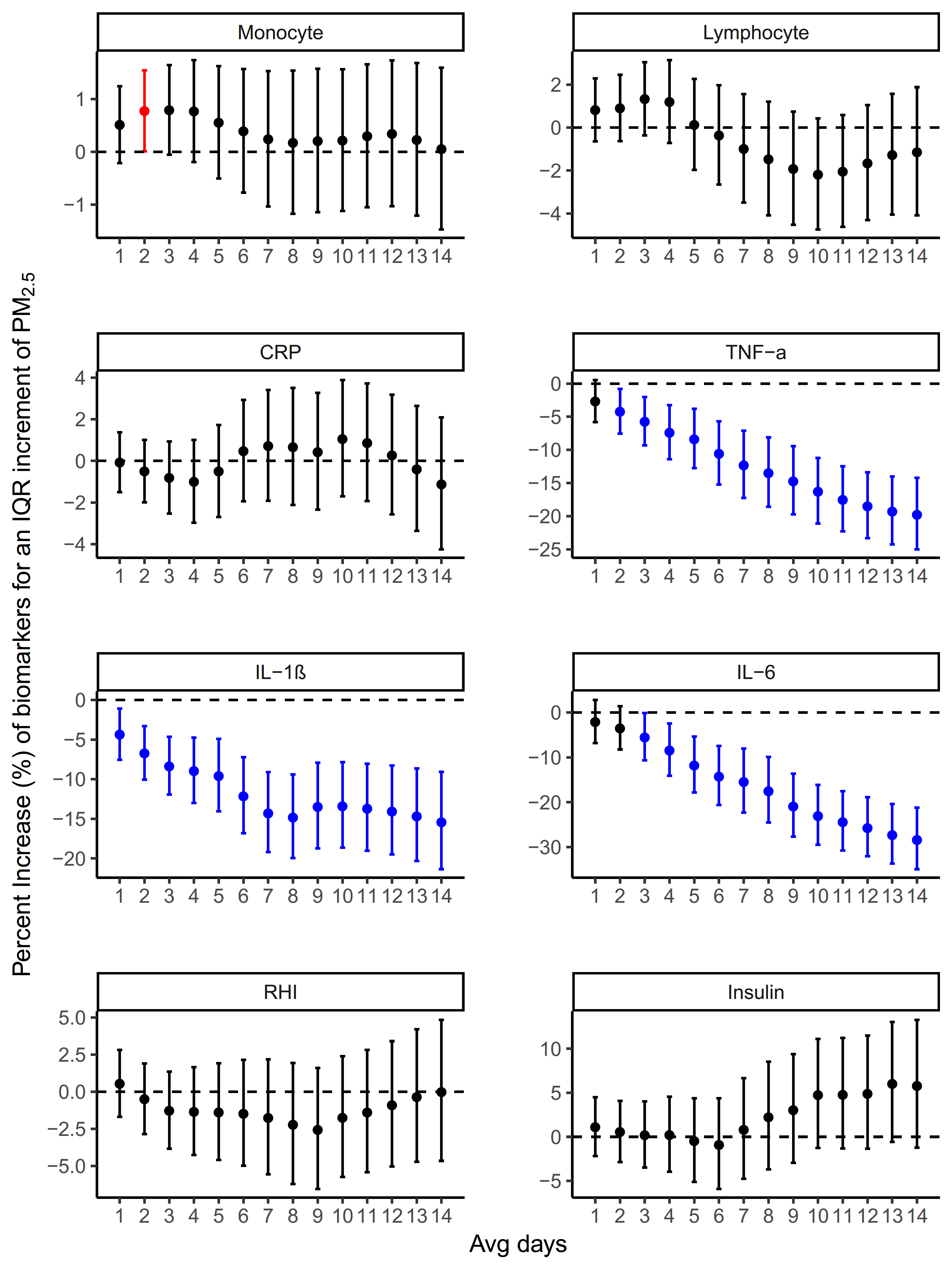


**Figure S4: Sensitivity analysis in nonsmokers: The ambient PM_2.5_ associated effect on monocytes, lymphocytes, CRP, Interlukin-1β, Interlukin-6, RHI and insulin in all the healthy and preDM subjects.**


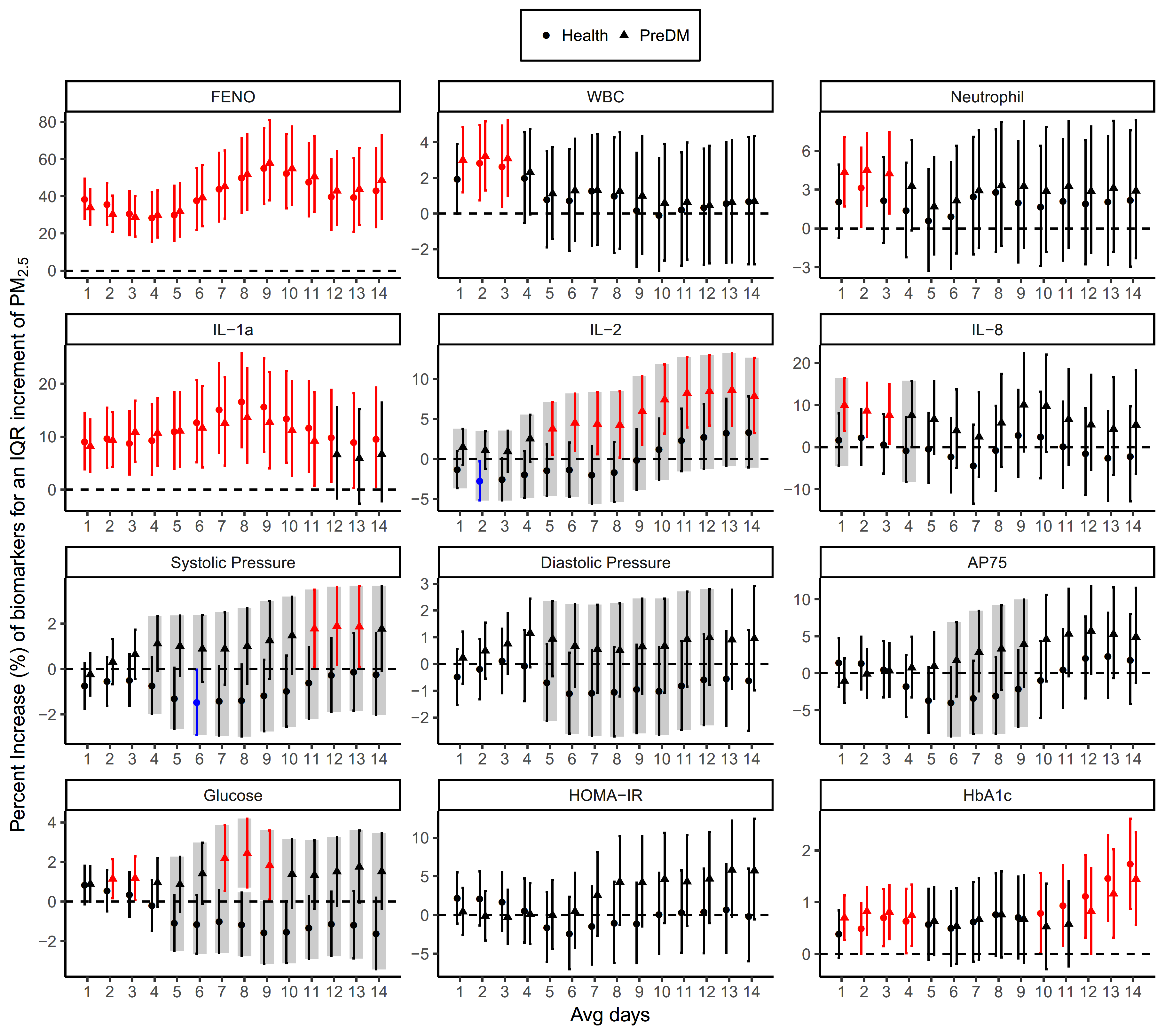
 **Figure S5: Sensitivity analysis in nonsmokers: Comparison of the ambient PM_2.5_ associated effect on 12 biomarkers (eNO, WBC, Neutrophil, Interlukin-1α, Interlukin-2, Interlukin-8, SBP, DBP, AP75, fasting glucose, HOMA-IR, and HbA1c) between the healthy and preDM subjects.**


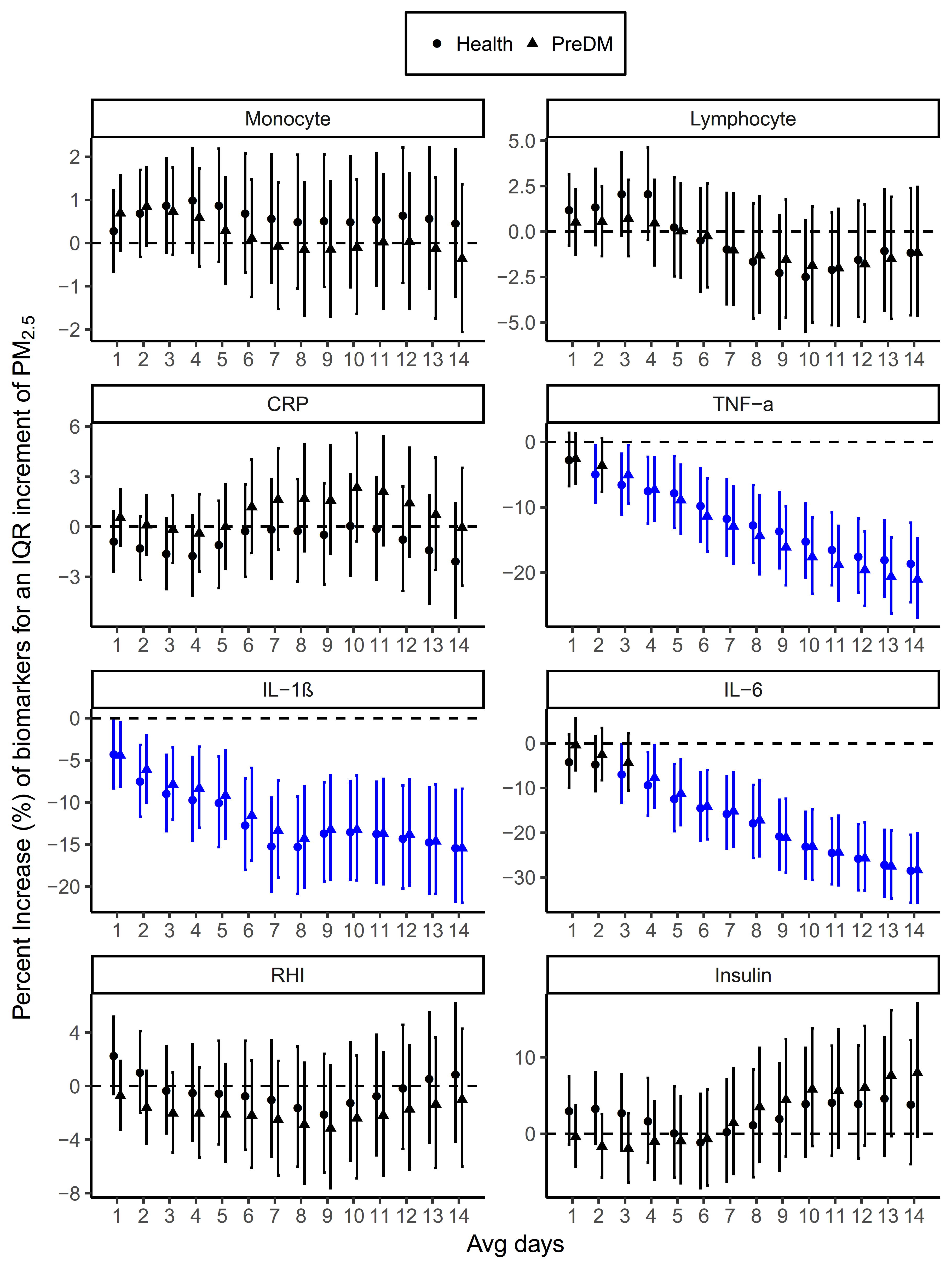


**Figure S6: Sensitivity analysis in nonsmokers: Comparison of the ambient PM_2.5_ associated effect on monocytes, lymphocytes, CRP, Interlukin-1β, Interlukin-6, RHI and insulin between the healthy and preDM subjects.**


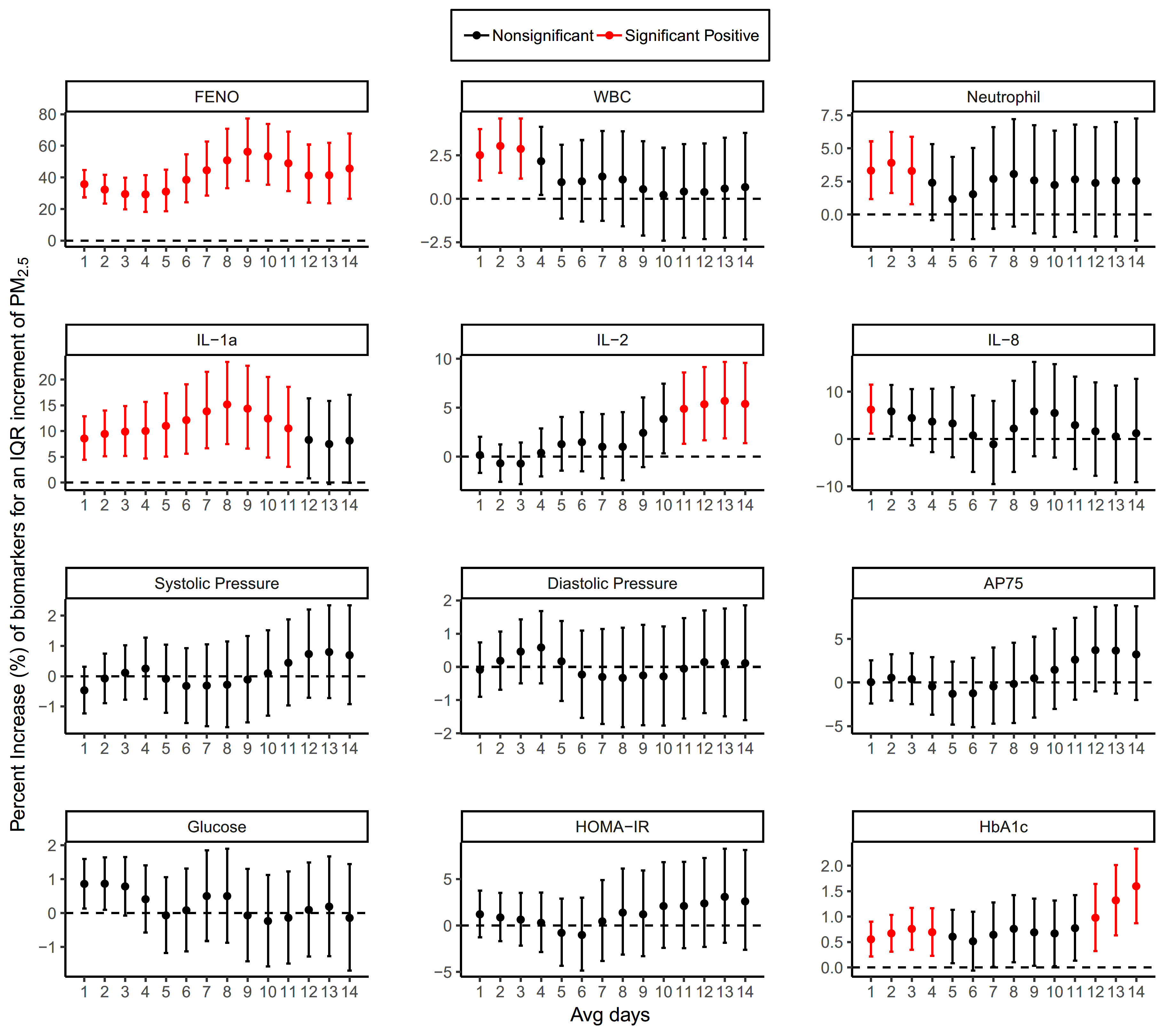


**Figure S7: False discovery rate (FDR) corrected ambient PM_2.5_ associated effect on 12 biomarkers (eNO, WBC, Neutrophil, Interlukin-1α, Interlukin-2, Interlukin-8, SBP, DBP, AP75, fasting glucose, HOMA-IR, and HbA1c) in all the healthy and preDM subjects.**


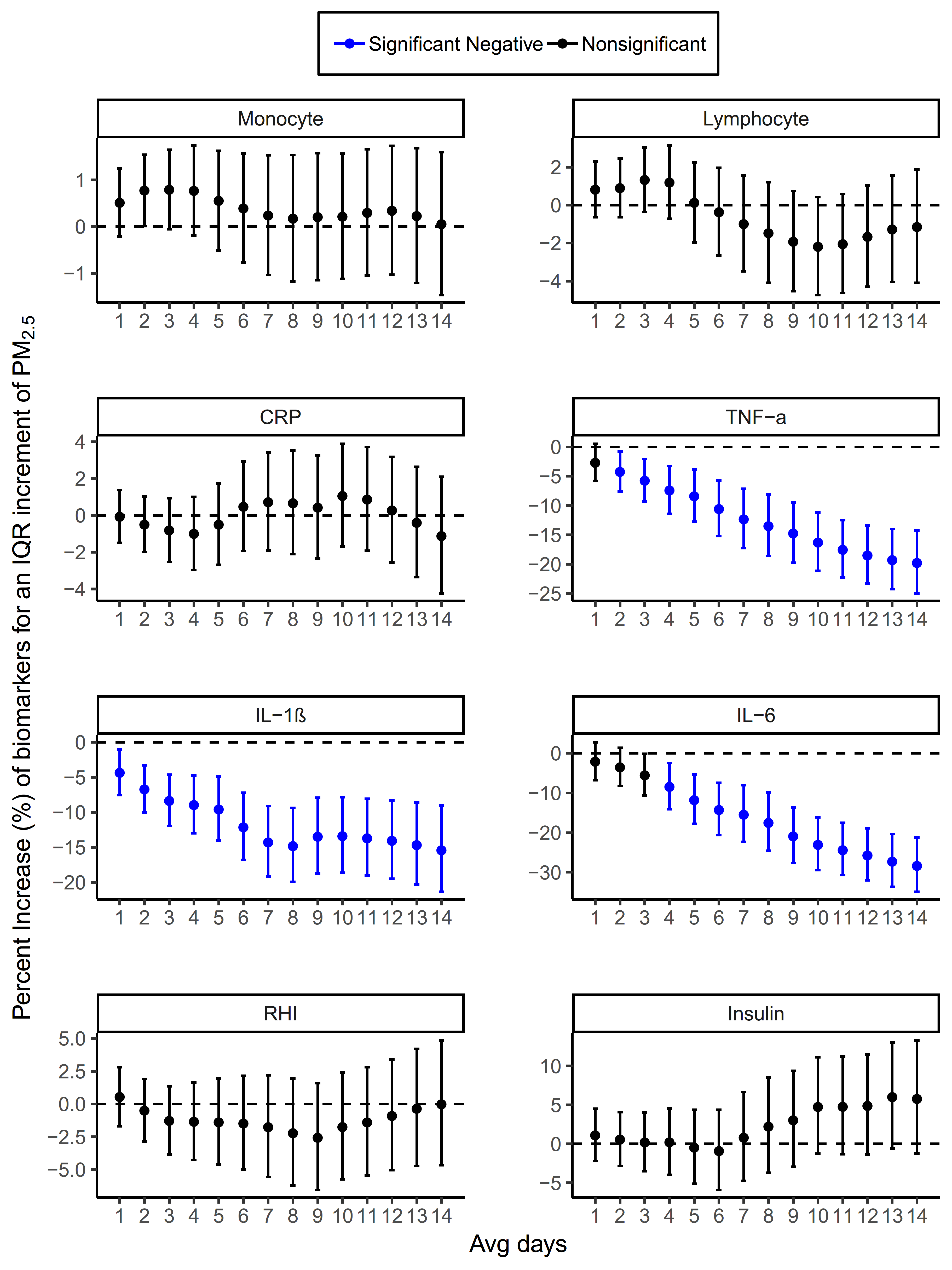


**Figure S8: False discovery rate (FDR) corrected ambient PM_2.5_ associated effect on monocytes, lymphocytes, CRP, Interlukin-1β, Interlukin-6, RHI and insulin in all the healthy and preDM subjects.**


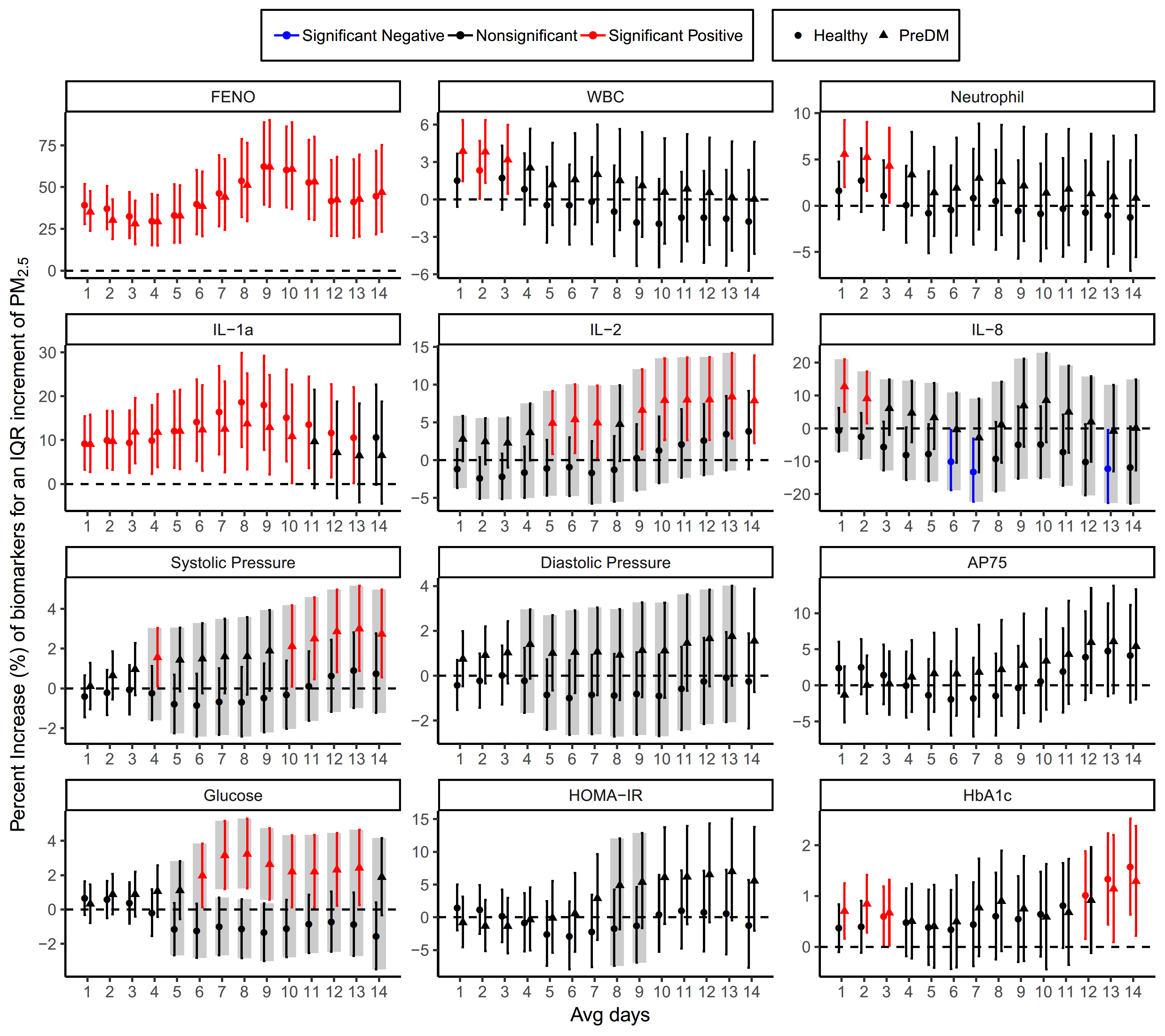


**Figure S9: Sensitivity analysis in normal tensive subjects: Comparison of the ambient PM_2.5_ associated effect on 12 biomarkers (eNO, WBC, Neutrophil, Interlukin-1α, Interlukin-2, Interlukin-8, SBP, DBP, AP75, fasting glucose, HOMA-IR, and HbA1c) between the healthy and preDM subjects.**
